# Acknowledgment to the Reviewers of *Infectious Disease Reports* in 2022

**DOI:** 10.3390/idr15010007

**Published:** 2023-01-19

**Authors:** Infectious Disease Reports Editorial Office

**Affiliations:** MDPI AG, St. Alban-Anlage 66, 4052 Basel, Switzerland

High-quality academic publishing is built on rigorous peer review. *Infectious Disease Reports* was able to uphold its high standards for published papers due to the outstanding efforts of our reviewers. Thanks to the efforts of our reviewers in 2022, the median time to first decision was 21 days and the median time to publication was 42 days. Regardless of whether the articles they examined were ultimately published, the editors would like to express their appreciation and thank the following reviewers for the time and dedication that they have shown *Infectious Disease Reports*:
Abdullah Salah AlanaziKalliopi MegariAbhijit A. AmbegaonkarKasi VegesanaAditya Yashwant SarodeKeita WagatsumaAgata MikolajczykKrishna C. ChintaAgnieszka TorzewskaLaura CapaAhmed MostafaLeo MassariAitor Garcia-VozmedianoLijun PeiAlan JacksonLuigia BruglieraAlessandra LatiniLuke AmbroseAlessandro N. VargasLuzia Helena QueirózAlessandro PerrellaMahrous Abdelbasset IbrahimAlexander D. BotvinkinMalik AydinAlexandre Pérez-GonzálezManoel Marques Evangelista De OliveiraAlexios BatrakoulisMarcia SaraivaAlfred O. AnkrahMarcie MiddlebrooksAlfredo CaturanoMarcin WełnickiAmrita SarkarMarco TjioeAna Del CerroMaria Angels ColomerAna Maria da Palma MoreiraMaria Rosaria CapobianchiAncy JosephMárió GajdácsAndrea BassiMark PeeplesAndrea CalcagnoMarta ArsuagaAndrea MarinoMarta CanutiAndrés Velasco-VillaMarta Torres-TorrillasAndrew ShelenkovMauro GiacomelliAnil Kumar VermaMauro PittirutiAnisuzzaman AnisuzzamanMd. Rabiul IslamAnna Kabłak-ZiembickaMelinda HerbathAnna MajewskaMichael BeetonAnna N. BerlinaMichael L. KuehtAnnika Helgadóttir DavidsenMichael MulemeAnthony J. KearsleyMinerva CruzAntje MunderMiri KrupkinAntonella CastagnaMirjana RadomirovicAntonella CingolaniMitsushige SugimotoAntonietta IvonaMohamed Hamdy YassinAntonio ZoccoliMohammad T. YousafzaiApurva Tadimari PrabhakarMonika FajferArlind MaraMrinal BhattacharjeeArmanda RodriguesMuhammad Hammad ButtArumugam Vijaya AnandMukesh VermaArun Saravanakumar AnnamalaiMurali MunirajuAsrat AmnieNadia SaklouAtefeh VaeziNamdev TogreAya SugiyamaNeeltje A. KootstraBahai JiaoNicola F. FletcherBan Hock TanNicole F. BernardBarbara BrognaNidhi SharmaBarbara DziegielewskaNiels Frimodt-MøllerBayodé Roméo AdégbitèNikola Zlatkov KolevBernhard ReschNina MendezBishara FreijNitin AmdareBruno ArcàNuno V. BritoCarla FontanaOlga CiepielaCarlo BieńkowskiOnder ErgonulCarmela MustoOwen LeiserCarmelo Ortega RodríguezPablo Jesús Marín-GarcíaCecilia PozziPalanivel ChinnakaliCecília R. C. CaladoPamela Di GiovanniCédric B. ChesnaisPaolo VersacciChaofan LiParag MaruChristian SiatkaParshva PatelCristina MesquitaPatrícia Aline Gröhs FerrarezeDaniela PierannunzioPaulo BettencourtDanijela ČernePeter SabakaDavid FrancoPhilip El-DuahDavid KrantzPierfrancesco GrimaDavid TurbowPiotr GolecDe Gaulle I. ChigbuPiotr MiśkiewiczDean KonjevićPrabhdeep KaurDeftereos SavasPrasanth BalasubramanianDelia GolettiPriya J. HalvorsenDi LiuRajdeep BanerjeeDianjun CaoRajkumar SahaniDimitra HouhoulaRedi RahmaniDivanshu ShuklaRegiane Maria Tironi De MenezesDomenico GalanteRegina Ferreira AlvesDominik ŁagowskiRegina Fölster-HolstDominik WawrzutaRenee L. GallowayEdward KrzyżakRicardo Sánchez De MadariagaEdyta Kaczorek-ŁukowskaRichard ParrishEhsan AhmadpourRitesh KumarEiichi MomotaniRodney P. JonesElena Marbán-CastroRodrigo Cuiabano Paes LemeElisa MazzottaRupinder KaurEman A. ToraihSabrina MartinezEmanuel Hernández-NúñezSabyasachi ChatterjeeEmmanouil KapetanakisSaggu Gagandeep SinghEwan PlantSandra KiazykFani LadomenouSanjay Kumar PatelFederica Dall'OglioSara Salehi HammerstadFeleke Hailemichael AstawesegnSayan Dutta GuptaFilippo FratiniSeema DangwalFlávia Venetucci GouveiaSeil KimFlora N. BalievaSenthil AthithanFrancesco Di GennaroSevero Vázquez PrietoFrancisco E. NicolásShailendra MauryaFrancisco Teixeira Da SilvaSheng FuFrank KrauseShigefumi OkamotoFred EyaseShihlung ChenFrederic SchoenbergShinya HasegawaGabriel D. PeckhamSilvia BianchiGabriela GoujgoulovaSimhachalam GurugubelliGaurav BairwaSimon PorcherGhazi KayaliSonthaya TiawsirisupGiacomo CasaliniSoren U. NielsenGianna DipalmaSotirios KakavasGilbert OctaviusSrijon BanerjeeGiovanna CalabreseSrinivas NellimarlaGiovanni BocciaStanislava IvanovaGiulia BarbieriStefania PerrucciGiuseppe PipitoneStefano Di BellaGrissell TiradoSubin Thenkunnel SurendranGuillaume GiraudSudipta BiswasGustavo FontechaSurajit ChatterjeeH. K. GohilSuyanto SuyantoHahn Young KimSuzy M. TeutschHao LiTaha MehanyHazuki TamadaTaiwo AremuHenri GruffatTanawin NopsoponHerbert Júnior DiasTaru SinghHoa Q. NguyenTerenzio CosioHsiao-Hsuan KuoTerrence J. RavineHyun-Woo ParkTorgny SunnerhagenIgor DumicTrevor L. BraselIleana Adela VacaroiuTrim LajqiIndu G. RajapakshaTusneaki KenzakaIzabela RaganUlrich SackJason Yiu Wing KamUrška RozmanJeffrey BaraUte RömlingJeffrey J. AdamoviczValeria TrivelloneJennifer RuddVan Giau VoJeremy L. GoodinVelasco CimicaJianping MaVeronica BarraganJisun LeeVictor Hugo C. de AlbuquerqueJoan HebdenVijay Singh GondilJoana DiasVinay ShivannaJoan-Francesc Fondevila-GascónVincenza GianfrediJoão Antônio Leal De MirandaWhitney A. QuallsJoão Manuel Graça FradeWilmer Gianfranco Silva-CasoJohad F. KhouryYash GuptaJosé Miguel Seguí-RipollYongfen XuJosé Rodrigo Da SilvaYugo AshinoJosue MartosYuyan WangJuan Alberto CorberaZaisen WangJuan ArciniegaZheng Zachory WeiJun InoueZhigang LyuKaixin LiangZhigang Qiu


